# Deficiencies of Inducible Costimulator (ICOS) During Chronic Infection with *Toxoplasma gondii* Upregulate the CD28-Dependent Cytotoxicity of CD8^+^ T Cells and Their Effector Function Against Tissue Cysts of the Parasite

**DOI:** 10.3390/cells13231998

**Published:** 2024-12-03

**Authors:** Rajesh Mani, Kanal E. Balu, Yasuhiro Suzuki

**Affiliations:** Department of Microbiology, Immunology and Molecular Genetics, University of Kentucky, Lexington, KY 40536, USA; rajesh.mani@uky.edu (R.M.); kanal_elamparithi.balu@uky.edu (K.E.B.)

**Keywords:** ICOS, CD28, costimulatory molecule, CD8^+^ T cell, cytotoxic activity, host defense, infection, *Toxoplasma gondii*

## Abstract

We recently identified that the cerebral mRNA expression of inducible costimulator (ICOS) and its ligand, ICOSL, both significantly increase during the elimination of *Toxoplasma gondii* cysts from the brains of infected mice by the perforin-mediated cytotoxic activity of CD8^+^ T cells. In the present study, we examined the role of ICOS in activating the effector activity of CD8^+^ T cells in response to the presence of cysts in infected mice. Following the adoptive transfer of splenic CD8^+^ T cells from chronically infected ICOS-deficient (ICOS^−/−^) and wild-type (WT) mice to infected SCID mice, fewer CD8^+^ T cells were detected in the brains of the recipients of ICOS^−/−^ CD8^+^ T cells than the recipients of WT CD8^+^ T cells. Interestingly, even with the lower migration rate of the ICOS^−/−^ CD8^+^ T cells, those T cells eliminated *T. gondii* cysts more efficiently than WT CD8^+^ T cells did in the brains of the recipient mice. Consistently, the ICOS^−/−^ CD8^+^ T cells secreted greater amounts of granzyme B in response to *T. gondii* antigens in vitro than WT CD8^+^ T cells did. We identified that CD8^+^ T cells of infected ICOS^−/−^ mice express significantly greater levels of CD28 on their surface than CD8^+^ T cells of infected WT mice, and the relative expression of CD28 mRNA to CD8β mRNA levels in the brains of the recipients of those CD8^+^ T cells were strongly correlated with their relative expression levels of mRNA for T-bet transcription factors and perforin. Furthermore, blocking CD28 signaling using a combination of anti-CD80 and anti-CD86 antibodies eliminated the increased cytotoxic activity of the ICOS^−/−^ CD8^+^ T cells in vitro. The present study uncovered notable compensatory interactions between ICOS and CD28, which protected the cytotoxic effector activity of CD8^+^ T cells against microbial infection in a murine model of chronic infection with *T. gondii*.

## 1. Introduction

*Toxoplasma gondii* is an obligate intracellular protozoan parasite that can infect all mammals, including humans [1,2]. This parasite forms tissue cysts in various organs, especially in the brain, and establishes a long-lasting chronic infection in these hosts [1,2]. Since tissue cysts can persist in immunocompetent hosts for long periods of time during the chronic stage of infection, it was previously thought that the immune system is unable to detect or attack the tissue cysts of this parasite. However, our recent studies uncovered that CD8^+^ T cells have the capability to detect the host cells harboring *T. gondii* cysts and destroy them through perforin-mediated effector activity [3,4]. Furthermore, we identified that CD8^+^ T cells penetrate into the cysts using their perforin-mediated activity and induce morphological deterioration and the destruction of the cysts, which is followed by the accumulation of large numbers of phagocytes [4]. When transcriptional levels for the immunity-related 734 molecules were compared in the brains of chronically infected SCID mice that had received CD8^+^ T cells from infected wild-type (WT) or perforin-deficient (Prf1^−/−^) mice, mRNA levels for only 6 molecules were identified to be significantly greater in the brains of the recipients of the WT CD8^+^ T cells than those of Prf1^−/−^ CD8^+^ T cells [5]. These six molecules were as follows: there were two T cell costimulatory molecules (inducible costimulator [ICOS] and its ligand [ICOSL]); two chemokine receptors (C-X-C motif chemokine receptor 3 [CXCR3] and CXCR6); and two molecules related to the activation of microglia and macrophages (interleukin 18 receptor 1 [IL-18R1] and chitinase-like 3) [5].

ICOS is a prominent costimulatory molecule that belongs to the CD28 receptor family. It supports the activities of both CD4^+^ and CD8^+^ T cells. However, in contrast to CD28, which is expressed in most T cells including naïve T cells, ICOS expression in CD8^+^ T cells is induced only after their activation through their T cell receptor engagement with target antigens presented by the MHC class I molecules [6,7]. In several bacterial and viral infections, the blocking or deficiency of ICOS resulted in reduced numbers of either the pathogen-specific [8,9] or IFN-γ-producing [10,11] CD8^+^ T cells. Reduced IFN-γ production and cytotoxic activity of CD8^+^ T cells were also observed in ICOS-deficient (ICOS^−/−^) mice infected with *Salmonella enterica* serovar Typhimurium [12]. In contrast, in persistent infection with *Plasmodium chabaude chabaude* AS, an intracellular protozoan parasite that proliferates within red blood cells, ICOS^−/−^ mice displayed increased numbers of IFN-γ^+^ CD8^+^ T cells [13]. Therefore, it is important to determine the role of ICOS specifically in the cytotoxic effector activity of CD8^+^ T cells against *T. gondii* cysts during chronic infection with this intracellular protozoan parasite.

To determine the effects of ICOS deficiency on the effector function of those CD8^+^ T cells to remove the tissue cysts of *T. gondii* from the brain tissue of infected hosts, in the present study, we transferred CD8^+^ immune T cells from infected wild-type (WT) and ICOS^−/−^ mice into infected SCID mice lacking T cells. Unexpectedly, we found that ICOS^−/−^ CD8^+^ T cells eliminated *T. gondii* cysts from the brains of the recipients more efficiently than WT CD8^+^ T cells, whereas fewer ICOS^−/−^ T cells migrated into the brains of the recipients than WT T cells. We identified that the ICOS^−/−^ CD8^+^ T cells expressed greater levels of CD28 on their surface than WT CD8^+^ T cells did. In addition, blocking the CD28 signaling pathway with a combination of anti-CD80 and anti-CD86 antibodies abolished the increased secretion of granzyme B (GzmB) by ICOS^−/−^ CD8^+^ T cells in response to the presence of *T. gondii* antigens in vitro, indicating that the upregulated expression of CD28 compensated for the absence of ICOS and maintained the cytotoxic effector activity of CD8^+^ T cells against *T. gondii* during chronic infection.

## 2. Materials and Methods

### 2.1. Mice

Female WT BALB/c, BALB/c-background ICOS^−/−^, and BALB/c-background SCID mice were obtained from the Jackson Laboratory (Bar Harbor, ME, USA). Outbred Swiss Webster mice were obtained from Taconic (Germantown, NY, USA). The studies were performed in accordance with the approved protocols (protocol #2020-3648) of the Institutional Animal Care and Use Committee of the University of Kentucky. There were 3 or 4 mice in each experimental group in each experiment.

### 2.2. Infection with T. gondii

Cysts of *T. gondii* were obtained from the brains of chronically infected Swiss Webster mice [14,15]. WT and ICOS^−/−^ mice were infected orally with 10 cysts by gavage and treated with sulfadiazine in drinking water (400 mg/L), beginning at 7 days after infection for 10 days, to assist in controlling tachyzoite proliferation during the acute stage of infection and to establish a chronic infection [14,15]. SCID mice were infected orally with 10 cysts by gavage and treated with sulfadiazine in the same manner. This began at 9 days after infection for the entire period of the experiments in order to maintain chronic infection in their brains [14,15].

### 2.3. Purification of CD8^+^ T Cells from Infected WT and ICOS^−/−^ Mice and Adoptive Transfer of the Purified T Cells into Infected SCID Mice

Spleen cells were obtained from ICOS^−/−^ and WT mice infected with *T. gondii* for at least 2 months. The cells were suspended in Hank’s balanced salt solution (HBSS) (HyClone [Cytiva], Mariborough, MA, USA) with 2% heat-inactivated fetal bovine serum (FBS) (Millipore-Sigma, Burlington, MA, USA). The spleen cells from four mice were pooled within the same experimental group, and CD8^+^ T cells were purified from the pooled spleen cell suspensions using magnetic bead-conjugated anti-mouse CD8α (clone 53-6.7) monoclonal antibodies (mAbs) (Miltenyi Biotech, Auburn, CA, USA) and an MACS column (Miltenyi) [14,15]. The high purity of the purified CD8^+^ T cells was confirmed via staining with FICT-labeled anti-mouse CD8 mAbs and APC-labeled anti-mouse CD3ε mAbs (BD Biosciences, San Jose, CA, USA). The purity of the CD8^+^ T cell preparations was consistently ≥95% (see Supplemental Figure S1 as an example). The purified CD8^+^ T cells were suspended in the 2% FBS-HBSS and injected intravenously from a tail vein into infected, sulfadiazine-treated SCID mice (2 × 10^6^ cells/mouse) 3 weeks after infection. Seven days later, the brain of each of the recipient mice was cut into halves, where one half was immediately frozen with dry ice for RNA purification and another half was fixed in a solution containing 10% formalin, 5% acetic acid, and 70% ethanol for immunohistochemical analyses. There were 3 or 4 mice in each experimental group, and two independent experiments were performed in the CD8^+^ T cell transfer study. Therefore, there were 7–8 mice in total in each experimental group.

### 2.4. RNA Purification and RT-PCR

RNA was purified from half of the brain of each mouse using RNA STAT-60 (Tel-test, Friendswood, TX, USA) and treated with DNase I (Invitrogen, Waltham, MA, USA) to remove genomic DNA contamination, as described previously [3,16]. cDNA was synthesized from either 1 or 4 μg of the DNase I-treated RNA from each brain sample. Quantitative PCR reactions were performed with the cDNA using StepOnePlus a real-time PCR system with Taqman reagents (Applied Biosystems, Branchburg, NJ, USA) [14,16]. The primers and probes for mouse β-actin (a house-keeping control molecule), CD8β, perforin, GzmB, CD28, 4-1BB (TNFRSF9), and T-bet were ready-made products acquired from Applied Biosystems. The primers and probes for bradyzoite (cyst)-specific BAG1 are as follows: 5′-TCACGTGGAGACCCAGAGT-3′ (forward), 5′-CTGGCAAGTCAGCCAAAATAATCAT-3′ (reverse), and 5′-TTTGCTGTCGAACTCC-3′ (probe) [16]. The amounts of mRNA levels for the targets of interest were normalized to amounts of mRNA for β-actin.

### 2.5. Immunohistochemistry

The fixed brains were embedded in paraffin, and sagittal sections (4 μm thickness) of the paraffin-embedded brains were stained for *T. gondii* or a combination of *T. gondii* and CD8^+^ cells. In *T. gondii* staining, the sections were stained with rabbit polyclonal anti-*T. gondii* antibodies, as previously described [4,16]. Dual staining for *T. gondii* and CD8α was performed using the Ventana Discovery Ultra instrument (Roche Diagnostics, Indianapolis, IN, USA). After deparaffinization and antigen retrieval with Ventana CC1 (Roche), the slides were incubated with rabbit polyclonal anti-*T. gondii* antibody at a 1:1000 dilution. This was followed by incubation with alkaline phosphatase-linked anti-rabbit IgG secondary antibody (Roche) and visualization with Discovery Red Chromogen (Roche). Residual antibodies were denatured by heating at 37 °C for 1 h using the CC2 antigen retrieval buffer prior to incubation with anti-CD8α antibodies at a 1:250 dilution (Cell Signaling Technology, Danvers, MA, USA), followed by incubation with Ventana anti-rabbit-HQ (Roche) for 20 min and Ventana anti-HQ-HRP (Roche). The staining was then amplified using Ventana’s Discovery TSA Amplification Kit (Roche) for 16 min. This was followed by linking with the Discovery Amplification Multimer-HRP (Roche) for 20 min and by DAB detection. Slides were counterstained with Meyer’s hematoxylin, blued, and permanently mounted. The cyst numbers in the entire field of each of the sagittal sections of the brains were microscopically determined. Three sections, with 16 or 20 μm distances between them, were assessed for each brain and the mean value of the counts from the three sections was used for each mouse. We microscopically counted CD8^+^ T cells in a total of 10 randomly selected fields at ×200 magnification in a sagittal section of each brain.

### 2.6. Culture of CD8^+^ T Cells Purified from Infected WT and ICOS^−/−^ Mice with T. gondii Antigens

After purifying CD8^+^ T cells from the spleens of chronically infected ICOS^−/−^ and WT mice using the anti-CD8-mAb-coated microbeads, the remaining CD8^−^ T cell-depleted spleen cells were suspended in an RPMI1640 medium (Gibco/Millipore Sigma, St. Louis, MO, USA) containing 10% FBS (HyClone [Cytiva]), 100 U/mL penicillin, and 100 μg/mL streptomycin (Invitrogen/ThermoFisher, Carlsbad, CA, USA). They were then cultured (5 × 10^5^ cells/well) in a flat-bottom 96-well tissue culture plate (Costar, Corning, Lowell, MA, USA) for 1.5–2 h. After the incubation, plastic non-adherent cells were removed by washing to prepare antigen-presenting cells (plastic adherent cells). Thereafter, CD8^+^ T cells purified from the spleens of those infected WT and ICOS^−/−^ mice were placed in those wells (3 × 10^5^ cells/well) containing the plastic adherent antigen-presenting cells from the corresponding strain of mice and cultured in the presence or absence of *T. gondii* tachyzoite lysate antigens (10 μg/mL) for 72 h [14,17]. In one experiment, we added blocking mAbs against ICOSL (clone HK5.3, BioLegend, San Diego, CA, USA), mAbs against CD80 and CD86 (clones 16-10A1 and PO3.1, respectively, Invitrogen/Thermo-Fisher), or a combination of these mAbs at 10 μg/mL to parts of these culture wells to block the ICOS-ICOSL or CD28-CD80/CD86, or both of these costimulatory pathways. As a control, isotype control mAbs were added in the same manner. There were 5 wells in each experimental group. The concentrations of GzmB in the culture supernatants were measured by ELISA using a commercial kit obtained from R&D Biosystems (Minneapolis, MN, USA) [17] by following their commercial inserts.

### 2.7. Flow Cytometry

CD8^+^ T cells purified from infected ICOS^−/−^ and WT mice were incubated with anti-Fcγ receptor monoclonal antibodies (mAbs) to block the antigen-nonspecific binding of mAbs to these cells. This was followed by incubation with FITC-labeled anti-CD8α and/or PE-labeled anti-CD28 for 30 min. As a control, these CD8^+^ T cells were incubated with FITC- or PE-labeled isotype control mAbs in the same manner. In a separate experiment, the purified CD8^+^ T cells were stained with FITC-labeled anti-CD8α, PE-labeled CD28, APC-labeled CD44, and APC-Cy7-labeled anti-CD62L mAbs. All of those antibodies were obtained from BD Biosciences. We used duplicated or triplicated tubes for each staining. The cells were analyzed with BD Symphony A3 using DIVA software version 9.1 (BD Biosciences, San Jose, CA, USA). The obtained data were further analyzed using FlowJo version 10.7.2 software (BD Biosciences). The purity of CD8^+^ T cells in the purified CD8^+^ T cell populations was consistently ≥95% (see Supplemental Figure S1).

### 2.8. Statistical Analysis

The levels of significance regarding the differences between experimental groups were determined by the Student’s *t* test or Mann–Whitney *U* test using GraphPad Prism software 9.0. The levels of significance in the correlations between two elements were determined by a Pearson or Spearman test using the same software. Differences that had *p* values < 0.05 were considered significant.

## 3. Results

### 3.1. CD8^+^ Immune T Cells from ICOS^−/−^ Mice Chronically Infected with T. gondii Have Increased Efficiency in Eliminating Tissue Cysts of the Parasite

**Figure 1:**
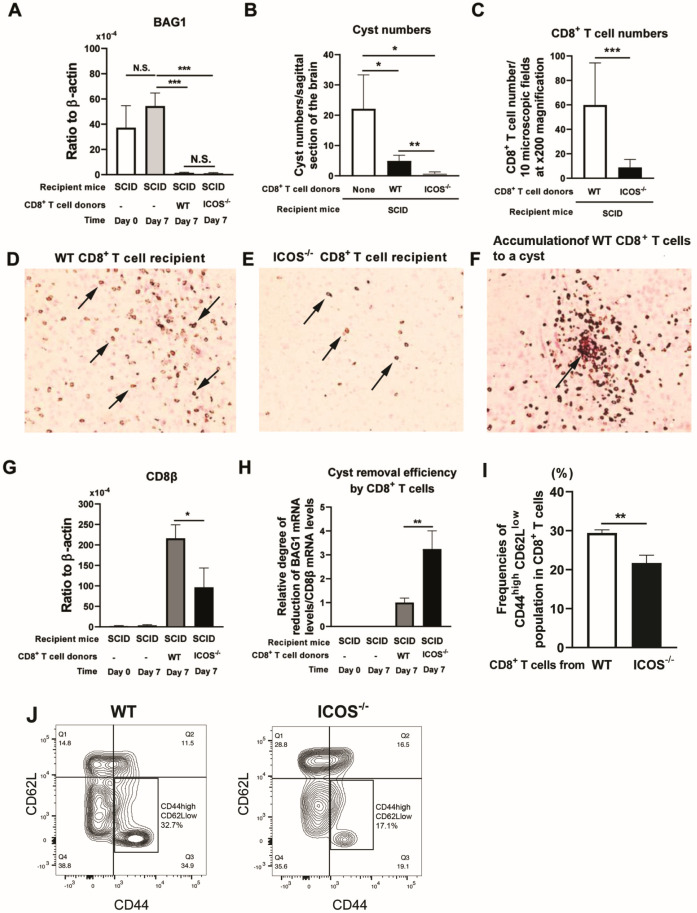
CD8^+^ immune T cells from ICOS^−/−^ mice chronically infected with *T. gondii* possess an increased capability to eliminate *T. gondii* cysts when compared to the T cells of infected WT mice. CD8^+^ T cells purified from the spleens of chronically infected WT and ICOS^−/−^ mice were injected (2 × 10^6^ cells/mouse) intravenously into chronically infected (infected and treated with sulfadiazine) SCID mice. As a control, two additional groups of the SCID mice did not receive any T cells. Seven days later (Day 7), the brains of the T cell recipients and one group of the control mice with no T cell transfer were obtained to measure mRNA levels for (**A**) bradyzoite (cyst)-specific BAG1 via RT-PCR. Brain samples from another group of the control mice with no T cell transfer were obtained on the day of the T cell transfer (Day 0) for the RT-PCR. (**B**) Numbers of *T. gondii* cysts per sagittal section of the brain on Day 7 in the sections immunohistochemically stained for the parasite. Three sections with 16 or 20 μm distance between sections were assessed for each mouse, and the mean value from the counts from the three sections was used for each mouse. (**C**) The CD8^+^ T cells in a total of 10 randomly selected fields at ×200 magnification of a sagittal section of the brain of each of the recipients of CD8^+^ T cells from ICOS^−/−^ or WT mice were counted microscopically after their immunohistochemical staining. (**D**) A representative image (×200 magnification) of CD8^+^ T cells (stained in brown, some are arrowed) detected in a sagittal section of the brains of WT CD8^+^ T cell recipients and (**E**) ICOS^−/−^ CD8^+^ T cell recipients. (**F**) A representative image (×200 magnification) of a *T. gondii* cyst (stained in red, arrowed) attacked by WT CD8^+^ T cells (stained in brown). (**G**) CD8β mRNA levels in the brains of the recipients of WT and ICOS^−/−^ CD8^+^ T cells. (**H**) The efficiency of cyst removal by CD8^+^ T cells that migrated into the brains of the recipients, which was calculated using the following formula: the ratios of BAG1 mRNA level reduction (the mean value of BAG1 mRNA levels [in ratio to β-actin mRNA levels] in the control mice with no T cell transfer at Day 7—BAG1 mRNA levels [in ratio to β-actin mRNA levels] in each of the recipients of WT or ICOS^−/−^ CD8^+^ T cells at Day 7) to the amounts of CD8β mRNA (in ration to β-actin mRNA levels) in the brain of each recipient mouse. There were four SCID mice in each of the groups that received WT or ICOS^−/−^ CD8^+^ T cells. (**I**) Frequencies of the CD44^high^CD62L^low^ effector memory population in the splenic CD8^+^ T cells of chronically infected ICOS^−/−^ and WT mice. (**J**) A representative FACS plot on the expression of CD44 and CD62L on CD8^+^ T cells from infected ICOS^−/−^ and WT mice. There were three or four SCID mice in the control group without any T cell transfer at each of Day 0 and Day 7. Regarding the donors of the CD8^+^ T cells, there were three or four mice in each of infected WT and ICOS^−/−^ mice, and their spleen cells were pooled within the same experimental group to purify CD8^+^ T cells. Two independent experiments were performed. Panels A, G, H, and I show the results obtained from the two independent experiments, which provided 7–8 mice in each experimental group. * *p* < 0.05; ** *p* < 0.01; *** *p* < 0.001; N.S., not significant.

To examine whether CD8^+^ T cells maintain their anti-cyst effector capability in the absence of ICOS during chronic infection with *T. gondii*, CD8^+^ T cells purified from the spleens of infected WT and ICOS^−/−^ mice were transferred to chronically infected (infected and treated with sulfadiazine) SCID mice. As a control, two additional groups of the infected SCID mice did not receive any T cells. High levels of bradyzoite (cyst)-specific BAG1 mRNA were detected in the brains of the control SCID mice with no T cell transfer at both the day of the transfer of CD8^+^ T cells (Day 0) and seven days after the T cell transfer (Day 7). In addition, their mRNA levels did not differ between these two time points (Figure 1A), indicating that the cyst burdens in the brains of these control mice were stable between these two time points. In contrast, seven days after the T cell transfer, BAG1 mRNA levels in the brains of the recipients of CD8^+^ immune T cells from the WT and ICOS^−/−^ mice were both more than 20 times lower than those of the control mice with no T cell transfer (*p* < 0.001, Figure 1A). In addition, the cerebral BAG1 mRNA levels in the recipients of ICOS^−/−^ CD8^+^ T cells were slightly lower than those in the recipients of WT CD8^+^ T cells (1.10 ± 0.49 vs. 1.47 ± 0.40 [×10^−4^] in BAG1 mRNA to β-actin ratios, Figure 1A), but this difference did not reach statistical significance.

We also examined the reduction in the cyst burdens of the recipients of the WT and ICOS^−/−^ CD8^+^ T cells by using immunohistochemical staining for *T. gondii* on their brains. Consistent with the BAG1 mRNA levels seen in these mice, the cyst numbers detected in the sagittal sections (three sections with 16–20 μm distances between sections) of the brains of these two groups of the CD8^+^ T cell recipients were markedly lower than those seen in the brains of the control mice with no T cell transfer (*p* < 0.05, Figure 1B). Notably, the cyst numbers in the recipients of the ICOS^−/−^ CD8^+^ T cells were significantly lower than those of the WT CD8^+^ T cell recipients (*p* < 0.01, Figure 1B).

We also performed immunohistochemical analysis to compare the numbers of CD8^+^ T cells detected in the brains of the recipients of WT and ICOS^−/−^ CD8^+^ T cells. We counted the numbers of CD8^+^ T cells detected in 10 randomly selected microscopic fields at ×200 magnification in a sagittal section of the brain of each of these recipient mice. The numbers of CD8^+^ T cells in the recipients of ICOS^−/−^ CD8^+^ T cells were markedly lower than those in the recipients of WT CD8^+^ T cells (*p* < 0.001, Figure 1C). Representative images of the CD8^+^ T cells detected in the immunohistochemically stained sections of the brains of each of these two groups of mice are shown in Figure 1D,E. The accumulation of CD8^+^ T cells to a *T. gondii* cyst detected in the brain of a recipient of WT CD8^+^ T cells is also shown in Figure 1F. These results indicate that although fewer ICOS^−/−^ CD8^+^ T cells were present in the brains of the recipient SCID mice than WT CD8^+^ T cells were at 7 days after their transfer to the recipients, the former reduced the cerebral cyst burdens in the recipients as efficiently as or even more efficiently than the latter during 7 days after their transfer to the recipient mice.

Consistent with the detection of fewer CD8^+^ T cells in the recipients of ICOS^−/−^ CD8^+^ T cells than those of WT CD8^+^ T cells, the amounts of CD8β mRNA in the brains of the former were less than half of what was seen in the latter (*p* < 0.05, Figure 1G). To further determine the efficiency of WT and ICOS^−/−^ CD8^+^ T cells that migrated into the brains of the recipient SCID mice in terms of eliminating *T. gondii* cysts from their brains, we calculated the ratios of BAG1 mRNA level reduction (the mean value of BAG1 mRNA levels [in ratio to β-actin mRNA levels] in the control mice with no T cell transfer at Day 7—BAG1 mRNA levels [in ratio to β-actin mRNA levels] in each of the recipients of WT or ICOS^−/−^ CD8^+^ T cells at Day 7) to the amounts of CD8β mRNA (in ratio to β-actin mRNA levels) in the brain of each recipient mouse. The cyst removal efficiency ratios in the brains of the ICOS^−/−^ CD8^+^ T cell recipients were 3.7 times greater than those of the WT CD8^+^ T cell recipients (*p* < 0.01, Figure 1H).

In regard to the presence of lower numbers of ICOS^−/−^ CD8^+^ T cells than WT CD8^+^ T cells in the recipient SCID mice, previous studies by others [18] and ourselves [19] identified that the CD8^+^ T cells which infiltrated into the brains of mice chronically infected with *T. gondii* belonged to the CD44^high^CD62L^low^ effector memory phenotype. Therefore, we examined whether the frequencies of the effector memory population in CD8^+^ T cells differ between the spleens of infected ICOS^−/−^ and WT mice. Notably, the frequencies of the CD44^high^CD62L^low^ effector memory population in the total splenic CD8^+^ T cell populations in infected ICOS^−/−^ mice were approximately 26% lower than those of infected WT mice (21.7 ± 2.00% vs. 29.4 ± 0.83%, *p* < 0.01, Figure 1I). A representative flow cytometric plot for the CD44 and CD62L staining of the ICOS^−/−^ and WT CD8^+^ T cells is shown in Figure 1J. The gating strategy employed in this flow cytometric analysis is shown in Supplemental Figure S2. The frequency of the CD44^high^CD62L^low^ effector memory population being lower in the ICOS^−/−^ CD8^+^ T cells than in the WT CD8^+^ T cells (Figure 1I) is consistent with the levels of CD8β mRNA seen in the brains of infected SCID mice that received the ICOS^−/−^ CD8^+^ T cells being lower than those of the recipients of WT CD8^+^ T cells, as shown in Figure 1G. Therefore, the low frequency of the CD44^high^CD62L^low^ effector memory population in CD8^+^ T cells in the spleens of infected ICOS^−/−^ mice is most likely a key factor that contributed to the presence of lower numbers of CD8^+^ T cells in the brains of the SCID mice that had received the splenic CD8^+^ T cells from the ICOS^−/−^ mice than in the recipients of the WT CD8^+^ T cells. Since our previous study showed that the CD44^high^CD62L^low^ effector memory population of CD8^+^ T cells migrates into the brains of *T. gondii*-infected mice through the interaction of α4β1 integrin, expressed on their surface, with VCAM-1, expressed on vascular endothelial cells [16], it is possible that most of CD8^+^ T cells other than the effector memory population do not express α4β1 integrin on their surface in *T. gondii*-infected mice. Therefore, they do not migrate into the brains when they are transferred into infected SCID mice.

### 3.2. ICOS^−/−^ CD8^+^ T Cells Express Greater Levels of mRNA for Perforin and T-Bet than WT CD8^+^ T Cells During Elimination of T. gondii Cysts in the Brain

**Figure 2:**
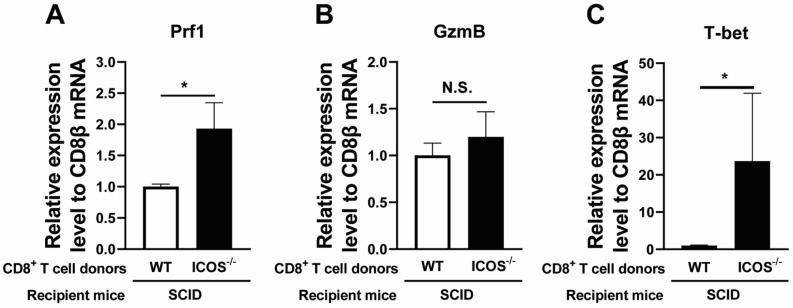
The relative mRNA expression levels of perforin, GzmB, and T-bet in ratio to CD8β mRNA levels are greater in the brains of SCID mice that received ICOS^−/−^ CD8^+^ T cells than those that received WT CD8^+^ T cells. CD8^+^ T cells purified from the spleens of chronically infected WT and ICOS^−/−^ mice were injected (2 × 10^6^ cells/mouse) intravenously into chronically infected (infected and treated with sulfadiazine) SCID mice. Seven days later (Day 7), the ratios of mRNA levels for (**A**) perforin, (**B**) GzmB, and (**C**) T-bet to mRNA levels to mRNA levels for CD8β were measured in the brains of those SCID mice by RT-PCR. There were four SCID mice in each of the groups. Two independent experiments were performed, and results from the two independent experiments were combined (a total of 8 mice in each experimental group). * *p* < 0.05, N.S., not significant.

Since perforin is required for the anti-cyst effector activity of CD8^+^ T cells [3,4], we compared the expression levels of mRNA for perforin in the ICOS^−/−^ and WT CD8^+^ T cells that had migrated into the brains of infected SCID mice after receiving the transfer of those T cells. For this purpose, we calculated the ratios of mRNA levels for perforin to mRNA levels for CD8β in the brains of those SCID mice at Day 7 after T cell transfer. The ratios of perforin mRNA levels to CD8β mRNA levels were twice greater in the recipients of ICOS^−/−^ CD8^+^ T cells than those in the recipients of WT CD8^+^ T cells (*p* < 0.05, Figure 2A). Since GzmB is another key effector molecule in the cytotoxic activity of CD8^+^ T cells, we also compared the ratios of mRNA levels for GzmB to mRNA levels for CD8β in the brains of the recipients of the ICOS^−/−^ and WT CD8^+^ T cells. The ratios of GzmB mRNA levels to CD8β mRNA levels were 24% greater in the recipients of ICOS^−/−^ CD8^+^ T cells than seen in the recipients of WT CD8^+^ T cells, but the difference did not reach statistical significance (Figure 2B). Since the transcription factor T-bet plays a critical role for the cytotoxic activities of CD8^+^ T cells [20,21,22], we also compared the ratios of T-bet mRNA levels to CD8β mRNA levels in the brains of the recipients of the ICOS^−/−^ and WT CD8^+^ T cells. These ratios were markedly greater in the recipients of ICOS^−/−^ CD8^+^ T cells than those of the recipients of WT CD8^+^ T cells (*p* < 0.05, Figure 2C). 

### 3.3. Increased CD28 mRNA Levels in ICOS^−/−^ CD8^+^ T Cells Correlate with Increased Levels of mRNA for T-Bet Transcription Factor for Their Cytotoxic Activity During Elimination of T. gondii Cysts in the Brain

**Figure 3:**
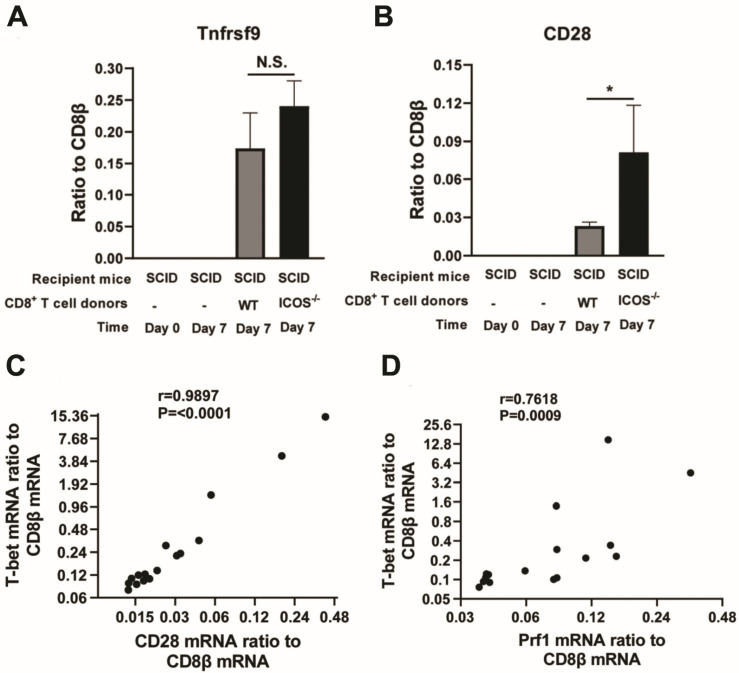
Relative mRNA levels for CD28, but not 4-1BB (TNFRSF9), are greater in ICOS^−/−^ CD8^+^ T cells that migrated into the brains of the recipient SCID mice than those of the WT CD8^+^ T cells that migrated into the brains of recipient SCID mice (**A**,**B**). Strong correlations are present between the increased relative mRNA expression levels of CD28 and those of T-bet (**C**) and between relative mRNA expression levels of T-bet and those of perforin (**D**) in the CD8^+^ T cells that migrated into the brains of the recipients during the elimination of *T. gondii* cysts. CD8^+^ T cells purified from the spleens of chronically infected WT and ICOS^−/−^ mice were injected (2 × 10^6^ cells/mouse) intravenously into chronically infected (infected and treated with sulfadiazine) SCID mice. Seven days later, their brains were obtained to measure (**A**) the ratios of 4-1BB (TNFRSF9) mRNA levels to CD8β mRNA levels, and (**B**) the ratios of CD28 mRNA levels to CD8β mRNA, by RT-PCR. The correlations of (**C**) the ratios of CD28 mRNA/CD8β mRNA levels with the ratios of T-bet mRNA/CD8β mRNA levels and (**D**) the ratios of T-bet mRNA/CD8β mRNA levels with the ratios of perforin mRNA/CD8β mRNA levels were examined in the brains of the recipients of the ICOS^−/−^ and WT CD8^+^ T cells. In these correlation analyses, the data from both the recipients of ICOS^−/−^ CD8^+^ T cells and those of WT CD8^+^ T cells were included. Two independent experiments were performed, and the results from these two experiments were combined (a total of 8 mice in each experimental group). * *p* < 0.05. N.S., not significant.

In addition to ICOS, CD28 and 4-1BB, expressed on the surface of CD8^+^ T cells, play important roles as costimulatory molecules in activating the T cells in response to target antigens [23]. Since CD8^+^ T cells of infected ICOS^−/−^ mice were found to have a significantly increased anti-cyst effector activity and perforin mRNA expression, it is possible that CD8^+^ T cells of the infected ICOS^−/−^ mice have increased expression levels of either 4-1BB or CD28, or both, to compensate for the absence of ICOS. To address this possibility, the ratios of mRNA levels for 4-1BB (TNFRSF9) and CD28 to CD8β mRNA levels were compared between the brains of the infected SCID mice that had received CD8^+^ T cells from infected ICOS^−/−^ and WT mice. Interestingly, the ratios of CD28 mRNA levels to CD8β mRNA levels were 3.7 times greater in the brains of the recipients of the ICOS^−/−^ CD8^+^ T cells than the recipients of the WT CD8^+^ T cells (*p* < 0.05, Figure 3B). The ratios of 4-1BB (TNFRSF9) mRNA levels to CD8β mRNA levels were also 43% greater in the brains of the recipients of ICOS^−/−^ CD8^+^ T cells than the recipients of WT CD8^+^ T cells, but the difference did not reach statistical significance (Figure 3A).

The transcription factor T-bet plays a critical role in the cytotoxic activities of CD8^+^ T cells [20,21,22], as mentioned earlier in Section 3.2. To address the possibility that costimulatory signals, mediated by the upregulated expression of CD28 in ICOS^−/−^ CD8^+^ T cells, contribute to the enhanced cytotoxic effector activity of those T cells in the removal of *T. gondii* cysts, we examined whether the increases in the ratios of CD28 mRNA levels to CD8β mRNA levels directly correlate with the increases in the ratios of T-bet mRNA levels to CD8β mRNA levels in the brains of the recipients of ICOS^−/−^ and WT CD8^+^ T cells. Notably, the degrees of increases in the CD28 mRNA/CD8β mRNA ratios strongly correlated with the degrees of increases in the T-bet/CD8β mRNA ratios in the brains of these recipient mice (*p* < 0.0001, Figure 3C). 

To support the possibility that the increased expression of transcription factor T-bet contributed to the upregulation of cytotoxic activity of ICOS^−/−^ CD8^+^ T cells, we examined whether the increases in the ratios of T-bet mRNA levels to CD8β mRNA levels directly correlated with increases in the ratios of perforin mRNA levels to CD8β mRNA levels in the brains of recipients of ICOS^−/−^ and WT CD8^+^ T cells. The degrees of increases in the ratios of T-bet mRNA levels/CD8β mRNA levels strongly correlated with the degrees of increases in the ratios of perforin mRNA levels/ CD8β mRNA levels in the brains of those T cell recipients (*p* = 0.0009, Figure 3D). The significant correlation in mRNA expression levels between CD28 and T-bet and between T-bet and perforin suggest that the costimulatory signal provided by upregulated expression of CD28 in ICOS^−/−^ CD8^+^ T cells contributes to their enhanced expression of transcription factor T-bet. The upregulated T-bet expression in those T cells then enhances their perforin expression and their perforin-mediated capability to eliminate *T. gondii* cysts from the brains of infected SCID mice that received these T cells.

### 3.4. Surface Expression of CD28 Costimulatory Factor Is Increased in ICOS^−/−^ CD8^+^ T Cells During Chronic Infection with T. gondii

**Figure 4:**
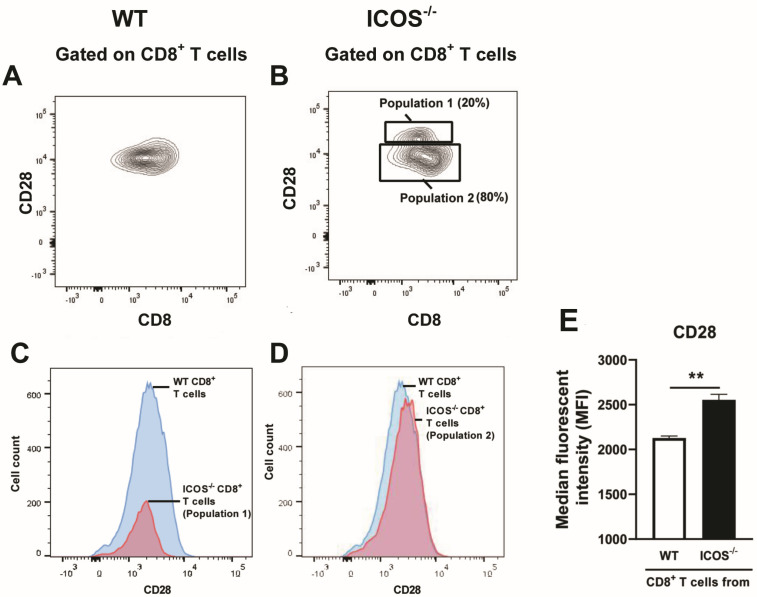
Splenic CD8^+^ T cells of ICOS^−/−^ mice chronically infected with *T. gondii* express greater levels of CD28 on their surfaces than CD8^+^ T cells of the infected WT mice. CD8^+^ T cells purified from the spleens of chronically infected WT and ICOS^−/−^ mice were stained with FITC-labeled anti-mouse CD8α and PE-labeled anti-mouse CD28 mAbs and applied for flow cytometric analysis. For control, the cells were stained with FITC- and PE-labeled isotype control mAbs. A representative image of the FACS plots for expressions of CD8 and CD28 on CD8^+^ T cells from chronically infected (**A**) WT and (**B**) ICOS^−/−^ mice is shown. Comparisons of CD28 expression levels between (**C**) the population 1 of ICOS^−/−^ CD8^+^ T cells (indicated in the panel **B**) and WT CD8^+^ T cells, and (**D**) between the population 2 of ICOS^−/−^ CD8^+^ T cells (indicated in the panel **B**) and WT CD8^+^ T cells, are shown. (**E**) The median fluorescence intensity (MFI) of CD28 expressions on the population 2 of ICOS^−/−^ CD8^+^ T cells and WT CD8^+^ T cells. ** *p* < 0.01.

CD28 provides the costimulatory signal when the molecule expressed on the surface of CD8^+^ T cells interacts with the ligands, CD80 and CD86, expressed on the surface of antigen-presenting cells, which present target antigens for the T cells. Based on the greater mRNA expression of CD28 in the ICOS^−/−^ CD8^+^ T cells than WT CD8^+^ T cells in the brains of recipients of these T cells, as shown in Figure 3B, we performed flow cytometric analyses to examine whether the expression levels of CD28 are increased on the surface of splenic ICOS^−/−^CD8^+^ T cells when compared to those levels of splenic WT CD8^+^ T cells during chronic *T. gondii* infection. Whereas WT CD8^+^ T cells displayed a single population based on their CD8 and CD28 expression levels (Figure 4A), the ICOS^−/−^ CD8^+^ T cells showed two populations that were distinct in terms of their expression levels of these two molecules (Figure 4B). In addition, the larger population (population 2, composing 80% of ICOS^−/−^ CD8^+^ T cells) of these two populations of ICOS^−/−^ CD8^+^ T cells had a significantly higher median fluorescent intensity (MFI) in its CD28 expression than the WT CD8^+^ T cells (*p* < 0.01, Figure 4D,E), although the MFI in CD28 expression in the smaller population (population 1, composing 20%) of ICOS^−/−^ CD8^+^ T cells was equivalent to that of WT CD8^+^ T cells (Figure 4C). These results indicate that the majority of splenic CD8^+^ T cells in chronically infected ICOS^−/−^ mice have increased expression levels of CD28 costmulatory factor on their surface when compared to those T cells in infected WT mice. At this moment, it is unclear why 20% (population 1) of the ICOS^−/−^ CD8^+^ T cells express lower CD28 expression levels than the rest (80%, population 2) of the CD8^+^ T cells do.

### 3.5. Blockage of CD28−CD80/CD86 Costimulatory Pathway Abolishes the Increased Cytotoxic Activity of CD8^+^ T Cells of ICOS^−/−^ Mice Chronically Infected with T. gondii

**Figure 5:**
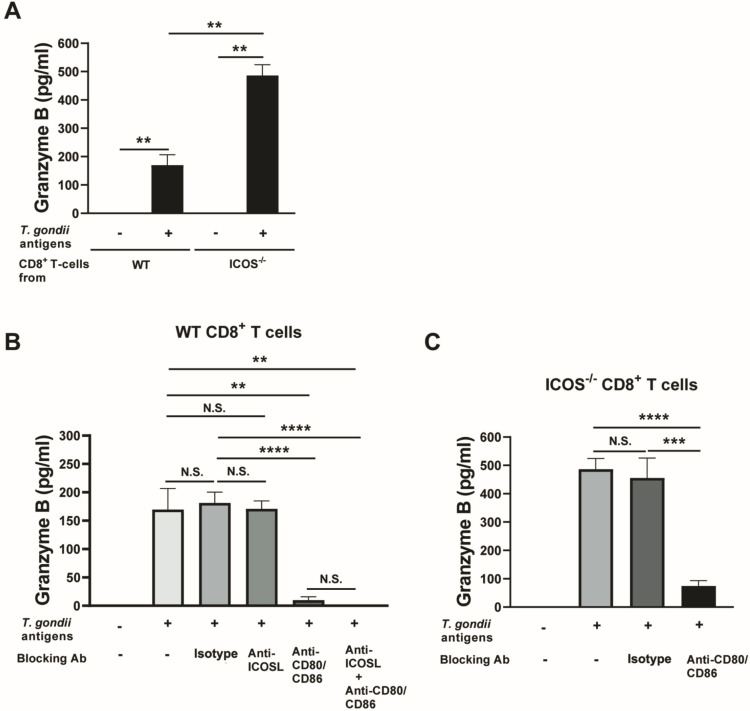
The blockage of the CD28−CD80/CD86 costimulatory pathway abolishes the cytotoxic functions of CD8^+^ T cells of ICOS^−/−^ mice chronically infected with *T. gondii*. The CD8^+^ T cells were purified from the spleens of chronically WT and ICOS^−/−^ mice and cultured (3 × 10^5^ cells/well) in 96-well culture plates with antigen-presenting cells (plastic-adherent cells) from the corresponding strain of mice in the presence or absence of *T. gondii* antigens (10 μg/mL) for 72 h. Blocking mAbs against ICOSL, both CD80 and CD86, or a combination of ICOSL, CD80, and CD86, were added at 10 μg/mL to a part of these cultures to block ICOS-ICOSL, CD28-CD80/CD86, or both of these costimulatory pathways. As a control, isotype control mAbs were added in the same manner. Concentrations of GzmB in the culture supernatants in the cultures were measured by ELISA. (**A**) A comparison of GzmB levels in the culture supernatants of WT and ICOS^−/−^ CD8^+^ T cells in the presence or absence of *T. gondii* antigens without any blocking mAbs. (**B**,**C**) The figures show comparisons of GzmB levels in the culture supernatants of (**B**) WT CD8^+^ T cells and (**C**) ICOS^−/−^ CD8^+^ T cells in the presence and absence of the blocking mAbs against the ICOS-ICOSL or CD28-CD80/CD86 costimulatory pathways. There were 2 mice in each of the infected WT and ICOS^−/−^ mice, and their spleen cells were pooled within the same experimental group to purify CD8^+^ T cells. There were 5 wells in each experimental group. ** *p* < 0.01, *** *p* < 0.001, **** *p* < 0.0001. N.S., not significant.

To further depict the increased cytotoxic activity of CD8^+^ T cells from infected ICOS^−/−^ mice against *T. gondii*, we compared the secretion of GzmB in response to the presence of *T. gondii* antigens between splenic ICOS^−/−^ and WT CD8^+^ T cells using in vitro cultures of these T cells. We also tried to measure the perforin levels of their culture supernatants, but we were unable to find a reliable ELISA kit for this purpose. After culturing the CD8^+^ T cells purified from the spleens of these mice with antigen-presenting cells (plastic adherent cells) from the corresponding mouse strain in the presence and absence of soluble *T. gondii* antigens for 72 h, the amounts of GzmB in the culture supernatants of the ICOS^−/−^ CD8^+^ T cells stimulated with the *T. gondii* antigens were found to be 5 times greater than those detected in the cultures of the WT CD8^+^ T cells stimulated with those antigens (*p* < 0.01, Figure 5A). In the absence of the *T. gondii* antigens in the cultures, the amounts of GzmB in the culture supernatants of both WT and ICOS^−/−^ CD8^+^ T cells remained very low and close to the detection limit of the ELISA assay (Figure 5A). The markedly increased secretion of GzmB from the ICOS^−/−^ CD8^+^ T cells, when compared to the WT CD8^+^ T cells, in response to *T. gondii* antigens was in contrast to the observation shown in Figure 2B, in which GzmB mRNA levels in the brains of the infected SCID mice that had received the ICOS^−/−^ CD8^+^ T cells did not significantly differ from those of the recipients of the WT CD8^+^ T cells. However, cytotoxic CD8^+^ T cells store pre-made cytotoxic enzymes such as GzmB within their intracellular granules and secrete these pre-made cytotoxic enzymes from those granules when they recognize their target cells [24,25]. Therefore, it is most likely that the increased secretion of GzmB by the ICOS^−/−^ CD8^+^ T cells when compared to the WT CD8^+^ T cells mostly involved the pre-made GzmB stored in their granules.

The binding of CD28 expressed on the surface of CD8^+^ T cells to CD80 and CD86 expressed on the antigen-presenting cells that present their target antigens provides the costimulatory signal required for the activation of those T cells, as mentioned earlier. To obtain direct evidence of the contribution of the upregulated CD28 expression in the CD8^+^ T cells of chronically infected ICOS^−/−^ mice to their upregulated cytotoxic activity against the parasite, we cultured the CD8^+^ T cells from infected ICOS^−/−^ and WT mice with antigen-presenting cells and *T. gondii* antigens in the presence and absence of blocking mAbs against CD80 and CD86. We also added anti-ICOSL mAbs to the cultures of WT CD8^+^ T cells to block the ICOS-ICOSL costimulation signal.

In cultures of WT CD8^+^ T cells, the blocking of CD28-CD80/CD86 costimulatory signaling by anti-CD80 and anti-CD86 mAbs markedly reduced the levels of GzmB in their culture supernatants (*p* < 0.0001, Figure 5B), whereas the addition of anti-ICOSL mAbs to these cultures did not affect the GzmB levels in their culture supernatants (Figure 5B). Although anti-CD80 and anti-CD86 antibodies could also block the inhibitory interaction of CTLA-4 with CD80/CD86 in CD8^+^ T cell activation, the blocking of this inhibitory pathway increases the activity of CD8^+^ T cells. Therefore, the significant decrease in GzmB secretion of CD8^+^ T cells seen due to treatment with the combination of anti-CD80 and anti-CD86 antibodies is mostly, if not all, due to the blocking of the CD28-CD80/CD86 costimulatory pathway. When both the ICOS and CD28 costimulatory signaling pathways were blocked by anti-ICOSL mAbs in combination with anti-CD80 and anti-CD86 mAbs, the levels of GzmB in the culture supernatants of WT CD8^+^ T cells stimulated with *T. gondii* antigens were as low as those of T cell cultures without stimulation with the parasite antigens (*p* < 0.0001 when compared to their cultures with isotype control mAbs in the presence of *T. gondii* antigens, Figure 5B). When comparing the cultures where there is only the blocking of the CD28 costimulatory pathway and cultures with the blocking of both CD28 and ICOS costimulatory pathways, GzmB levels in the former tended to be higher than in the latter, but this difference did not reach statistical significance (Figure 5B). These results indicate that the presence of CD28 costimulatory signaling without ICOS costimulatory signaling can provide the costimulatory signaling required for the activation of the cytotoxic activity of WT CD8^+^ immune T cells of chronically infected mice in response to their target *T. gondii* antigens, although the absence of both CD28 and ICOS costimulatory signaling seems to be required to completely block the cytotoxic activity of CD8^+^ T cells from chronically infected WT mice in response to *T. gondii* antigens. 

In the cultures of ICOS^−/−^ CD8^+^ T cells, the blocking of CD28 costimulatory signaling by anti-CD80 and anti-CD86 mAbs markedly (6.4 times) reduced the levels of GzmB in their culture supernatants in response to *T. gondii* antigens (*p* < 0.001, Figure 5C). This is consistent with the observations from WT CD8^+^ T cells, in which the presence of CD28 costimulation signaling without ICOS costimulation pathway can provide sufficient costimulatory signaling to efficiently activate the cytotoxic activity of the CD8^+^ T cells in response to *T. gondii* antigens, whereas the absence of both costimulatory pathways ablated the cytotoxic activity in response to target antigens. Thus, these results indicate that the increased cytotoxic activity of ICOS^−/−^ CD8^+^ T cells against *T. gondii* antigens is mediated by their increased expression of CD28. These results also depicted important compensatory interactions between the two costimulatory pathways, which were mediated by ICOS and CD28 and helped to secure the cytotoxic effector functions of CD8^+^ T cells against *T. gondii* in order to overcome the absence of ICOS by utilizing the increased CD28 expression. 

## 4. Discussion

The present study using the adoptive transfer of CD8^+^ T cells from ICOS^−/−^ and WT mice chronically infected with *T. gondii* to infected SCID mice revealed that a deficiency of ICOS during chronic infection with this parasite increases the efficiency of the CD8^+^ T cells in terms of eliminating tissue cysts of this parasite from the brains of the recipients. Our previous studies identified that CD8^+^ T cells eliminate *T. gondii* cysts using their perforin-dependent cytotoxic activity [3,4]. Consistently, the present study also revealed that the relative expression levels of perforin mRNA in ratio to CD8β mRNA in the brains of the recipients of the ICOS^−/−^ CD8^+^ T cells are significantly greater than those of the recipients of the WT CD8^+^ T cells, suggesting that the ICOS^−/−^ CD8^+^ T cells express greater levels of perforin mRNA in the brains of the recipient SCID mice than the WT CD8^+^ T cells do. Furthermore, the present study identified that CD8^+^ T cells from infected ICOS^−/−^ mice secrete much greater amounts of GzmB in response to *T. gondii* antigens in vitro than CD8^+^ T cells from infected WT mice do. GzmB is a key effector molecule, in addition to perforin, in the cytotoxic activity of CD8^+^ T cells. In relation to our findings, a recent study with persistent infection with *Plasmodium chabaude chabaude* AS, an intracellular protozoan parasite closely related to *T. gondii*, demonstrated that infected ICOS^−/−^ mice displayed increased numbers of IFN-γ^+^ CD8^+^ T cells when compared to infected WT mice [13]. In contrast, previous studies using infections with viruses [10,26] and bacteria [8,11,12] showed that an absence of ICOS costimulatory signaling activity, induced by either the genetic deletion of ICOS or the blocking of its functions by anti-ICOS mAbs or ICOS-Ig (a fusion protein of ICOS and the Fc region of human IgG1), downregulates [8,10,11,12] or does not affect [26] the cytotoxic activity and/or IFN-γ production of CD8^+^ T cells during those microbial infections. The present study provides new insights, showing that ICOS deficiency induces the upregulation of cytotoxic activity and the effector function of CD8^+^ T cells against *T. gondii* cysts during chronic infection with this protozoan parasite. Therefore, the effects of the absence of ICOS costimulatory activity on the functions of CD8^+^ T cells during microbial infections most likely differ depending on the types of pathogens. 

The present study was performed during the chronic stage of *T. gondii* infection, in which WT and ICOS^−/−^ mice were infected for at least 2 months. During the acute stage of infection, *T. gondii* tachyzoites resides and actively proliferate within the parasitophorous vacuole (PV) in infected host cells. The PV prevents fusion with lysosomes and protects the parasite from their elimination [27,28]. Similarly, *Mycobacterium tuberculosis,* an intracellular bacterium, also resides within phagosomes in infected cells, and prevents the fusion of those phagosomes with lysosomes [29,30,31]. In infection with *M. tuberculosis*, the bacterial loads in the spleen did not differ between ICOS^−/−^ and WT mice during the first 40 days of the infection, but the pathogen loads become significantly less in the former than the latter at 60 and 120 days after infection [11]. Of interest, the significantly reduced bacterial loads in the ICOS^−/−^ mice during the later time points of the infection are associated with increased numbers of IFN-γ^+^ CD4^+^ T cells in the spleens of these mice [11]. Therefore, the effects of ICOS deficiency on CD4^+^ and CD8^+^ T cells could differ depending on the time periods that the hosts were infected with certain pathogens. 

There are notable differences in resistance and susceptibility to chronic infection with *T. gondii* among inbred strains of mice [32,33,34]. Mice with the H-2^b^ (e.g., C57BL/6) and H-2^k^ haplotypes (e.g., C3H/He) are susceptible to and develop progressive and ultimately fatal toxoplasmic encephalitis during the later stage of infection, whereas mice with the H-2^d^ haplotype (e.g., BALB/c) are resistant and maintain a latency of the chronic infection in their brains [32,33,34]. The present study was performed in the genetically resistant BALB/c-background mice. A previous study, conducted by others [35] using the BALB/c-background ICOS^−/−^ mice, showed that whereas percentages of IFN-γ^+^ cells in CD4^+^ T cells in the spleens were reduced in ICOS^−/−^ mice when compared to WT mice during the acute stage (day 7) of *T. gondii* infection, percentages of IFN-γ^+^ cells in CD8^+^ T cells did not differ between ICOS^−/−^ and WT mice in their spleens during the early stage of infection and in their brains during a later stage (weeks 4–6) of infection. In contrast, the present study revealed greater cytotoxic effector activity of CD8^+^ T cells against *T. gondii* cysts in BALB/c-background ICOS^−/−^ than seen in WT mice during the chronic stage of infection. Therefore, it may be possible that the IFN-γ production and cytotoxic activity of CD8^+^ T cells are controlled in a different manner through ICOS-mediated pathways.

In contrast to the genetically resistant BALB/c mice, mice with genetically susceptible C57BL/6-background showed that the blocking of ICOS signaling by anti-ICOSL mAbs or the genetic deletion of ICOS increases numbers of CD4^+^ and CD8^+^ T cells and IFN-γ^+^ CD8^+^ T cells in the spleens and brains 5–6 weeks after infection, but significantly greater numbers of *T. gondii* cysts were detected in the brains of the infected ICOS^−/−^ than WT mice [36,37]. In the present study on genetically resistant BALB/c-background mice, we identified that numbers of ICOS^−/−^ CD8^+^ T cells in the brains of the SCID mice that had received those T cells were smaller than those in the brains of the recipients of WT CD8^+^ T cells. In addition, our study revealed that the ICOS^−/−^ CD8^+^ T cells eliminated *T. gondii* cysts from the brains of the recipients more efficiently than the WT CD8^+^ T cells did. It is most likely that the roles of ICOS in the protective activities of CD8^+^ T cells against *T. gondii* differ depending on the genetic resistance and susceptibility of the hosts to the infection.

Previous studies, conducted using infections with vaccinia virus [38], influenza virus [9], and *Listeria monocytogenes* [39], demonstrated the requirement of CD28 for optimal recall responses of CD8^+^ T cells. Notably, the present study, conducted using flow cytometry, identified that the absence of ICOS is compensated by the upregulation of CD28 expression levels in splenic CD8^+^ T cells during the chronic stage of *T. gondii* infection. The present study also consistently identified that the ratios of CD28 mRNA levels to CD8β mRNA levels in the brains of infected SCID mice that had received splenic CD8^+^ T cells from infected ICOS^−/−^ mice were significantly greater than those in the recipients of the CD8^+^ T cells from infected WT mice. Furthermore, the in vitro stimulation of splenic CD8^+^ T cells of infected ICOS^−/−^ and WT mice with *T. gondii* antigens revealed that upregulated CD28 expression mediates the increased cytotoxic effector activity of the ICOS^−/−^ CD8^+^ T cells in their recall responses to the pathogen.

The transcription factor T-bet plays a critical role in the cytotoxic activities of CD8^+^ T cells [20,21,22]. The present study identified that the degrees of increases in relative expression levels of mRNA for CD28 in ratio to CD8β mRNA levels strongly correlate with the degrees of increases in ratios of T-bet mRNA levels to CD8β mRNA levels in the brains of the recipients of the ICOS^−/−^ and WT CD8^+^ T cells. In addition, the degrees of increases in the ratios of T-bet mRNA levels to CD8β mRNA levels in the brains of the recipients of those CD8^+^ T cells strongly correlated with the degrees of increases in the ratios of perforin mRNA levels to CD8β mRNA levels in the brains of those mice. Therefore, the costimulatory signal, enhanced through the increased expression of CD28 in ICOS^−/−^ CD8^+^ T cells, most likely induced the upregulation of perforin mRNA levels through the increased expression of T-bet transcription factor and thereby enhanced the efficiency of the elimination of *T. gondii* cysts through their cytotoxic activity. To our knowledge, the upregulation of CD28 expression in CD8^+^ T cells in compensation for a deficiency of ICOS and an enhancement of their cytotoxic effector activity through the upregulated CD28 expression have not been reported before.

In relation to our findings on the compensation for the absence of ICOS via the upregulation of CD28 expression in the cytotoxic activity of CD8^+^ T cells in their recall responses to *T. gondii* antigens, a previous study with infections with lymphocytic choriomeningitis virus and vesicular stomatitis virus demonstrated that the blocking of ICOS signaling by ICOS-Ig markedly impaired IFN-γ production of CD4^+^ T cells against the viruses in CD28^−/−^ mice, whereas ICOS-Ig treatment in WT mice had only a limited downregulatory effect on IFN-γ production [26]. Thus, it is possible that there is not only compensation for the absence of ICOS by the upregulation of CD28 expression but also compensation for the absence of CD28 by the upregulation of ICOS, which can be used to maintain the effector functions of not only CD8^+^ T cells but also CD4^+^ T cells during microbial infections. Similarly, a previous study conducted by others [40] using *T. gondii* infection demonstrated that the blocking of the ICOS costimuratory pathway by an anti-B7RP-1 antibody inhibited IFN-γ production by splenocytes of infected CD28^−/−^ mice in response to the parasite antigens in vitro, and that the treatment of CD28^−/−^ mice with the anti-B7RP-1 antibody led to their increased mortality during the acute acquired stage of the infection. They did not examine IFN-γ production of CD4^+^ or CD8^+^ T cells in infected CD28^−/−^ mice and did not compare ICOS expression levels in CD4^+^ or CD8^+^ T cells between infected CD28^−/−^ and WT mice. In the present study, we discovered the occurrence of significantly increased expressions of CD28 on CD8^+^ T cells in *T. gondii*-infected ICOS^−/−^ mice when compared to infected WT mice during the chronic stage of the infection. We also found that the increased CD28 expression of the ICOS^−/−^ CD8^+^ T cells mediates a marked upregulation of their cytotoxic activity against the parasite. Therefore, it is possible that, in a previous study by others [40], the ICOS expression in CD4^+^ and/or CD8^+^ T cells increased in the infected CD28^−/−^ mice when compared to the infected WT mice, and that their increased ICOS expression induced increased IFN-γ production in the CD28^−/−^ mice in a similar manner as was discovered in infected ICOS^−/−^ mice.

The present study provided novel insights on the notable capability of the immune system to secure the protective activities of CD8^+^ T cells by utilizing compensatory interactions between two important costimulatory molecules, ICOS and CD28, to enhance host resistance during chronic infection with *T. gondii*. The results of the present study may also suggest that, in the presence of CD28 expression, the increased expression of ICOS in WT CD8^+^ T cells, as detected in our recent study [5], could enhance their anti-cyst effector activity, allowing them to eliminate *T. gondii* cysts.

## 5. Conclusions

This work showed that the absence of ICOS during chronic infection with *T. gondii* induces the significant upregulation of the expression of another costimulatory molecule, CD28, but not 4-1BB, in CD8^+^ T cells. It also showed that the upregulated CD28 expression mediates the increased cytotoxic effector activity of the CD8^+^ T cells, which is required to eliminate tissue cysts of the parasite from the brains of chronically infected mice. Indeed, the present study further revealed that CD8^+^ T cells of infected ICOS^−/−^ mice have an increased capability to eliminate *T. gondii* cysts when compared to the T cells of infected WT mice. To our knowledge, the upregulation of CD28 expression in CD8^+^ T cells in compensation for a deficiency in ICOS and the enhancement of the cytotoxic effector activity of the T cells through the upregulated CD28 expression, have not been reported before. Thus, the present study shed light on notable compensatory interactions between ICOS and CD28, which secure the cytotoxic effector activity of CD8^+^ T cells against a microbial infection, in a murine model of chronic infection with *T. gondii*.

## Data Availability

The data generated from this study are presented in this article. Further inquiries can be directed to the corresponding author.

## Supplementary Materials

The following supporting information can be downloaded at: https://www.mdpi.com/article/10.3390/cells13231998/s1, Figure S1: A representative flow cytometric plots displaying high purities of CD8^+^ T cells purified from the spleens of WT and ICOS^−/−^ mice chronically infected with *T. gondii*. CD8^+^ T cells purified from the spleens of WT and ICOS^−/−^ mice chronically infected with *T. gondii* using magnetic beads-conjugated anti-mouse CD8α (clone 53-6.7) mAbs and MACS column were stained with FITC-labeled anti-CD8α, and APC-labeled anti-CD3ε mAbs; Figure S2: The gating strategy applied to the flow cytometric analyses for expressions of CD44 and CD62L on CD8^+^ T cells in the spleens of WT and ICOS^−/−^ mice chronically infected with *T. gondii*. The spleen cells from the infected WT and ICOS^−/−^ mice were stained with FITC-labeled anti-CD8α, PE-labeled CD28, APC-labeled CD44, and APC-Cy7-labeled anti-CD62L mAbs. (A) The gating for live lymphocytes. (B) The gating for CD8^+^ T cells. Those CD8^+^ T cell populations in the infected WT and ICOS^−/−^ mice were further applied for their expressions of CD44 and CD62L shown in Figure 1J.
