# Supplementary material for: Deficiencies of Inducible Costimulator (ICOS) During Chronic Infection with Toxoplasma gondii Upregulate the CD28-Dependent Cytotoxicity of CD8+ T Cells and Their Effector Function Against Tissue Cysts of the Parasite
Source: Cells. 2024 Dec 3;13(23):1998. doi: 10.3390/cells13231998 (PMC11640114; doi:10.3390/cells13231998)
Supplement: Supplementary file 1 [file cells-13-01998-s001.zip › cells-3239073-supplementary.pdf]

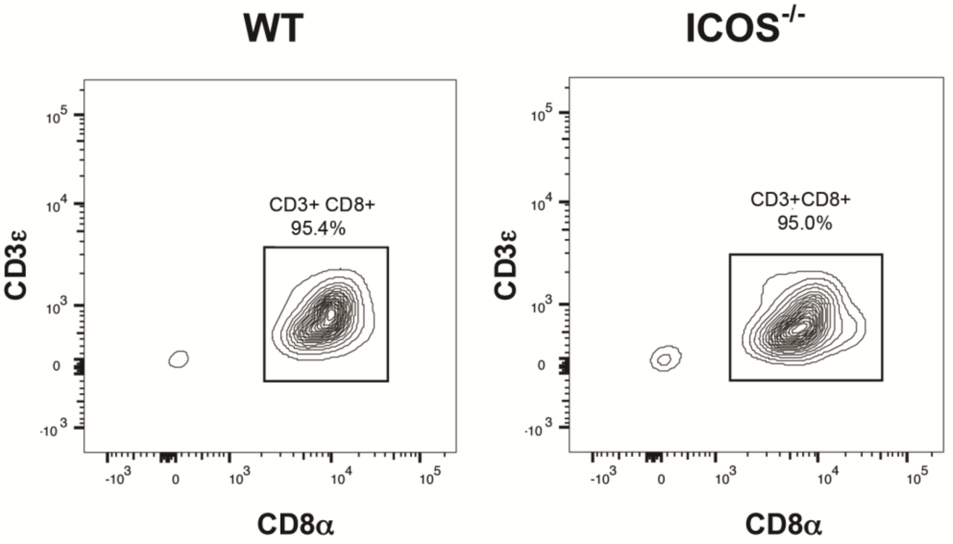

Supplemental Fig. S1. A representative flow cytometric plots displaying high purities of $CD8^+$ T cells purified from the spleens of WT and $ICOS^{-/-}$ mice chronically infected with *T. gondii*. $CD8^+$ T cells purified from the spleens of WT and $ICOS^{-/-}$ mice chronically infected with *T. gondii* using magnetic beads-conjugated anti-mouse CD8α (clone 53-6.7) mAbs and MACS column were stained with FITC-labeled anti-CD8α, and APC-labeled anti-CD3ε mAbs.

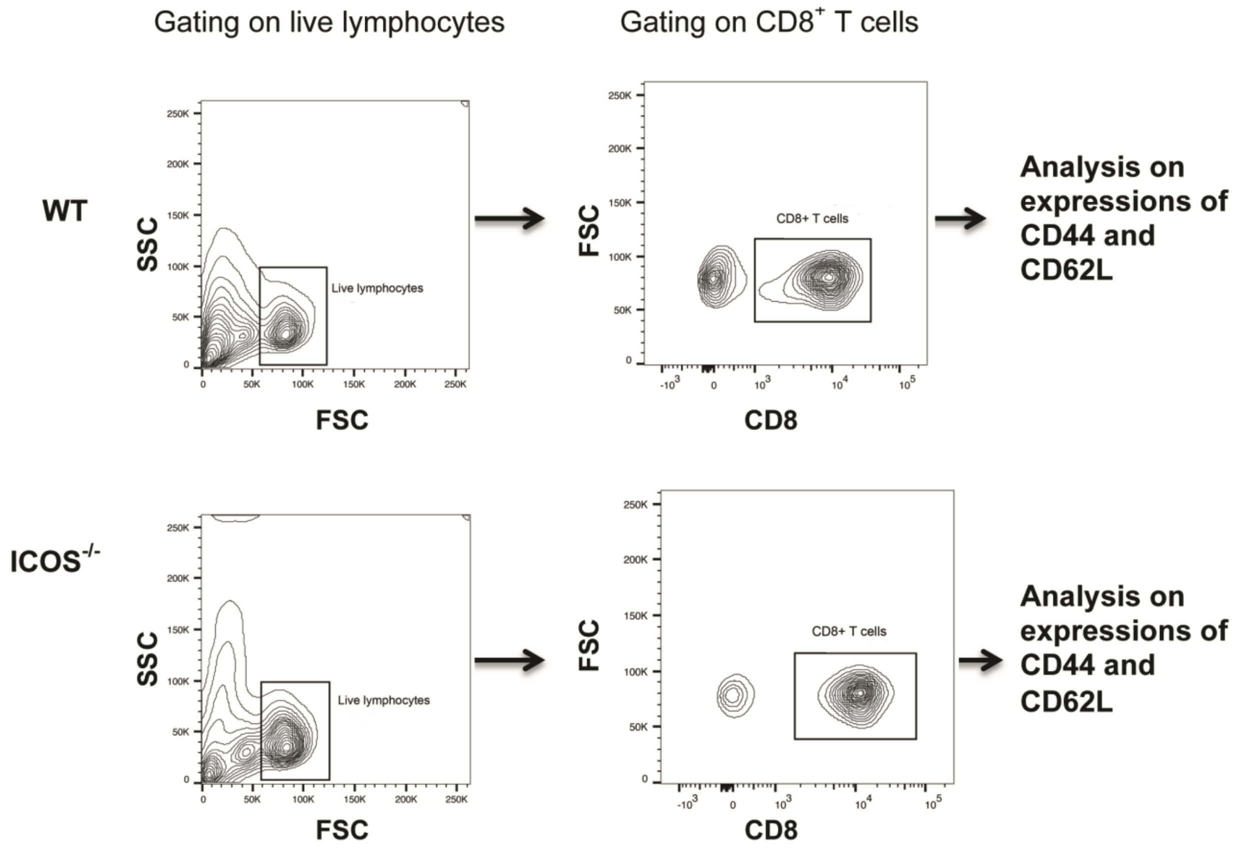

Supplemental Fig. S2. The gating strategy applied to the flow cytometric analyses for expressions of CD44 and CD62L on $CD8^+$ T cells in the spleens of WT and $ICOS^{-/-}$ mice chronically infected with *T. gondii*. The spleen cells from the infected WT and $ICOS^{-/-}$ mice were stained with FITC-labeled anti-CD8α, PE-labeled CD28, APC-labeled CD44, and APC-Cy7-labeled anti-CD62L mAbs. (A) The gating for live lymphocytes. (B) The gating for $CD8^+$ T cells. Those $CD8^+$ T cell populations in the infected WT and $ICOS^{-/-}$ mice were further applied for their expressions of CD44 and CD62L shown in Fig. 1J.
